# Overview of the Current State of Flexible Solar Panels and Photovoltaic Materials

**DOI:** 10.3390/ma16175839

**Published:** 2023-08-25

**Authors:** Rashid Dallaev, Tatiana Pisarenko, Nikola Papež, Vladimír Holcman

**Affiliations:** Department of Physics, Faculty of Electrical Engineering and Communication, Brno University of Technology, Technická 2848/8, 616 00 Brno, Czech Republic; 177722@vut.cz (T.P.); papez@vut.cz (N.P.); holcman@vut.cz (V.H.)

**Keywords:** solar panels, photovoltaics, perovskite-based, silicon-based, flexible

## Abstract

The rapid growth and evolution of solar panel technology have been driven by continuous advancements in materials science. This review paper provides a comprehensive overview of the diverse range of materials employed in modern solar panels, elucidating their roles, properties, and contributions to overall performance. The discussion encompasses both traditional crystalline silicon-based panels and emerging thin-film technologies. A detailed examination of photovoltaic materials, including monocrystalline and polycrystalline silicon as well as alternative materials such as cadmium telluride (CdTe), copper indium gallium selenide (CIGS), and emerging perovskite solar cells, is presented. Furthermore, the impact of transparent conductive materials, encapsulation polymers, and antireflective coatings on solar panel efficiency and durability is explored. The review delves into the synergistic interplay between material properties, manufacturing processes, and environmental considerations. Through a comprehensive survey of materials utilized in modern solar panels, this paper provides insights into the current state of the field, highlighting avenues for future advancements and sustainable solar energy solutions.

## 1. Introduction

From the first half of the 19th century to the present day, the topic of photovoltaics has been enriched by theoretical and practical research. French physicist Alexandre Edmond Becquerel discovered the photovoltaic effect in 1839. Fundamental research was continued in 1873 by Willoughby Smith (conductivity of selenium), and Charles Fritz in 1883 created the world’s first module of selenium elements. Then followed the discovery by the German physicist Heinrich Rudolf Hertz in 1887 of a new phenomenon: “external photoelectric effect”. From 1888 to 1891, Alexander G. Stoletov successfully continued further research in this area. The theoretical justification for the photoelectric effect was made by Albert Einstein in 1907. The acute demand for semi-conductor cells has expanded and accelerated research in the field of engineering and photovoltaics. In 1916, Polish scientist Jan Czochralski discovered the processes of crystal formation by growing single crystals from a melt. Experiments were carried out with a thin film of copper oxide (1934) and with silicon-based samples (1940), which were marked by the discovery of the p-n junction in semiconductors; the theoretical model of the p-n junction by William Bradford Shockley laid the foundation for the development of modern solar cells. Daryl Chapin, Calvin Fuller, and Gerald Pearson introduced crystalline silicon solar cells in 1953.

Numerous research teams are presently engaged in photovoltaics research across global universities and research establishments. This research can be categorized into three domains: enhancing the cost-effectiveness and efficiency of contemporary solar cells to establish their competitive stance among alternative energy sources, innovating new technologies through novel architectural approaches for solar cells, and advancing novel materials that enhance the conversion of light energy into electric current or act as superior absorbers and conductors of light-induced charges.

In this regard, this particular review paper seeks to provide a comprehensive and up-to-date examination of the current state of flexible solar panels and photovoltaic materials. In particular, the focus is on elucidating the intricate relationship between the materials employed in solar panels, their inherent properties, the roles they play within the photovoltaic system, and their profound influence on the overall performance of flexible solar panels.

With a growing array of materials being explored for photovoltaic applications, ranging from traditional silicon-based semiconductors to emerging organic, perovskite, and thin-film materials, understanding the nuances of each material’s characteristics has become pivotal. This review aims to bridge the knowledge gap by systematically analyzing and comparing the key attributes of various materials, including their electronic properties, bandgap engineering, charge transport mechanisms, and stability. By delving into the fundamental science behind these materials, we aim to provide readers with a clear understanding of how material selection shapes the efficiency, durability, and adaptability of flexible solar panels.

Furthermore, the roles of encapsulation layers, substrates, and transparent conductive materials are equally paramount in determining the performance and reliability of flexible solar panels. This is why the aim is also to explore how these components contribute to enhancing light absorption, electron-hole separation, and overall device stability, while also addressing challenges such as mechanical flexibility, weather resistance, and cost-effectiveness.

Ultimately, by examining the intricate interplay between photovoltaic materials and panel design, this review aspires to equip researchers, engineers, and policymakers with a comprehensive survey of the current landscape. It aims to foster a deeper appreciation for the materials-driven advancements in flexible solar panels and highlight the pivotal role that material innovation plays in shaping the trajectory of solar energy technology. As the world continues to transition toward renewable energy solutions, a nuanced understanding of these materials and their influence on solar panel performance will undoubtedly accelerate the realization of a sustainable and resilient energy future.

This comprehensive review paper meticulously assembles and evaluates an extensive spectrum of data drawn from diverse sources, encompassing scientific literature, cutting-edge research articles, and insightful industry reports. By amalgamating these diverse resources, the review offers an all-encompassing elucidation of the prevailing landscape concerning flexible solar panels and photovoltaic materials.

## 2. Current State, Market Shares, and Future Outlook

The rapid development of solar energy, using innovative world technologies, is the main competitor, and in 2050 it will be predominant in the market for energy-friendly technologies, which will cover all the electric energy needs of the population by the end of the century [1]. The annual amount of solar energy coming to the Earth is 1018 kWh, while the land surface accounts for about 20% of this energy. The energy characteristics of photocells are mainly determined by the following parameters: the intensity of solar radiation, the magnitude of the load, and the operating temperature [2]. Since the distance from the Earth to the Sun changes during the year within 150 million km, the amount of solar radiation also varies from 1325 to 1420 W/m^2^ [3].

One of the promising areas for solving the problem of energy supply to consumers is the development and implementation of renewable energy sources (RES). Their share of world consumption in 2020 was around 24% and according to forecasts this number will increase by ~50% by the year 2040 [3].

The dynamically developing solar energy complex puts forward a number of tasks for all economic entities. The main function of the solar energy complex (SEC) is the production and redistribution of energy obtained through the use of solar energy. The production of heat and electricity is based on two main technologies that make it possible to divide the solar energy complex into two subtypes: photovoltaic SEC and solar thermal SEC. Photovoltaic SEC uses technologies for direct conversion of solar energy into electricity, and solar thermal SEC uses technologies for converting solar energy into heat (using solar collectors) and technologies for concentrating solar energy for its subsequent conversion into electricity [4,5,6,7,8].

Solar power plants have long been an integral part of the energy balance of the largest economies in the world. According to IRENA, over the past 10 years the installed capacity of solar power plants in the world has grown more than 17 times, from 41.6 GW to 714 GW. At the same time, 127 GW of new capacities were installed in 2020 alone. The production of solar panels in the world is also growing steadily. According to the American consulting company Clean Energy Associates (CEA), the global capacity for the production of solar panels themselves by the end of 2021 reached about 400 GW, and the capacity for the production of new elements for panels is 325 GW [9].

From 1999–2008 the main contribution to the development of solar energy was made mainly by four countries: Japan, Germany, the USA, and Spain. Since 2004, the volume of annual installed capacities has increased dramatically in Germany, and it has done so since 2007 in Spain [10].

By the end of 2008, the installed capacity of solar panels worldwide reached 15 GW, of which 65% are located in Europe, 15% in Japan, and 8% in the USA. With record growth in installed capacity in Spain (from 560 MW in 2007 to 2511 MW in 2008) and notable growth in installations in Italy (from 42 MW in 2007 to 258 MW in 2008) and South Korea (from 43 MW in 2007 to 274 MW in 2008) 2008 was a unique year in the development of solar energy [10].

In 2006, the first solar installations appeared in Belgium (2 MW), France (8 MW), and Greece (1 MW); in 2007, they appeared in the Czech Republic (3 MW) and Portugal (14 MW). In one or two years, these European countries, as well as the leaders, increased the volume of annual installations many times over. Thus, in 2008, 48 MW of solar panels were already installed in Belgium, 46 MW in France, 11 MW in Greece, 51 MW in the Czech Republic, and 50 MW in Portugal. Such a noticeable increase in installation volumes is explained by the introduction of special bonus tariffs for solar electricity [10].

Germany accounted for the lion’s share of the European PV market in 2010 with 7.408 GW of installed capacity, equivalent to 56%. The second country in terms of installed capacity in Europe in 2010 was Italy with 2321 GW or 18% of the market share. The Czech Republic had a fairly significant share: 11% and 1.49 GW of installed capacity by 2010. France closes the top five European market leaders with 719 MW of installed capacity, which is 5% [11].

The growth of solar energy in Europe once again surpassed all analysts’ forecasts. Despite stagnation in many sectors of the economy and resistance to the further implementation of large solar projects in some countries (for example, in the Czech Republic), 21,939 GW of solar installations were connected to the grid on the continent, which is almost 1.7 times more than in 2010 (13 GW) [12].

For the first time in history, Italy became the leader in terms of installed new capacity (9.3 GW), leaving behind Germany with 7.5 GW. It should be noted that the joint share of Italy and Germany amounted to more than 60% in the total installed capacity in 2011 in Europe. They are followed by France (1.7 GW), Belgium (974 MW), and the UK (784 MW) [11].

Analyzing the segmentation of the European photovoltaic market, one should note the predominance of the share of large ground-based solar stations in countries such as Bulgaria, Romania, Slovakia, and Spain (more than 85% of the cumulative capacity). Solar panels in the residential sector are dominated by the Czech Republic (over 60%), the Netherlands (about 70%), and Denmark (about 98%). In Sweden, Austria, Hungary, and Slovenia, the predominance of the commercial sector is noticeable (more than 50%) [13].

Numerous studies in the field of photovoltaics to find the ideal formula for the production of solar cells with higher efficiency and low cost have found a design solution in creating convenient and environmentally friendly flexible (folding) solar panels of various modifications.

The global solar photovoltaic (PV) market has been growing since 2005 by an average of 40% per year. This is far more than any other industry [14].

Solar PV is expected to create more than 2 million jobs in the next 20 years and reduce greenhouse gas emissions into the atmosphere by 350 million tons of CO_2_, which is equivalent to stopping 140 coal-fired power plants. The total solar PV capacity in 2030 will exceed 650 GW [15,16].

If at the early stages of the development of solar energy at the regional level it could be described as monocentric, now the emergence of new growth poles is an active process. In North America, Canada joined the “solar club”, which until recently included only the United States, Europe, Great Britain, and Belgium, who were then joined by Germany, Italy, France, and Spain. In the future, Bulgaria and the Czech Republic may become leaders. Of the traditional European centers for the development of the industry, Germany, Italy, and Spain still retain their importance. Over the past 10 years, the Asian world center has been actively developing, led by China, Japan, and India. Along with the dynamic development of large centers, there is a mass of less significant ones, which are nonetheless contributing to a change in the structure of the location of industry facilities. These new centers are likely to become locomotives for the development of global solar energy. The main global trend is the preservation of leadership for Europe and the gradual spread of the industry in North America and Asia. In the countries of South America and Africa, solar energy has not yet become so widespread, but in the future these countries represent some of the main regions for the development of the industry [17].

At the end of 2017, the installed capacity of global solar PV exceeded 400 GW and covered approximately 2% of global electricity demand. More than 90% of the current global production of modern solar photovoltaic panels use wafer-based crystalline silicon technology [18].

Most flexible solar panels are used at solar stations operating in various climatic zones, regardless of weather conditions. Experts predict by 2040–2050 the transition from rigid modifications to a new generation of “solar films” will occur.

Attention is drawn to the excellent mechanical properties of polymers, their ability to be processed and the variety of their forms and derivatives, as well as their high absorption coefficient in the optical range, which allows for their use in the form of ultra-thin (several hundred nanometers) films deposited from solutions at ordinary pressure on flexible substrates with unlimited area. This makes it possible to manufacture polymer solar cells (SC) using cheap mass production methods such as inkjet printing and stamping [19].

To provide mankind with energy in 2050, it is necessary to generate up to 71 TW of electricity annually. The only technology that can solve this problem is roll-to-roll technology: the technology of flexible printed electronics. Two of the leaders in the development of flexible polymer and composite solar cells are Konarka Technologies Inc. and Solarmer Energy Inc. (USA). At Solarmer Energy Inc., in 2011, the highest efficiency of a tandem polymer SC~8.13% was achieved, and at Konarka Technologies Inc., in February 2012, a record-breaking efficiency of single-layer polymer SCs was obtained and remains so to date at 9% (on laboratory samples) [20].

Such studies in the development of solar cells based on optically active conjugated polymers and polymer–inorganic nanoparticle composites, which are multilayer polymer and composite structures in which the layers consist of various polymers and nanoparticles, are conducted at the Organic Electronics Group of the Ioffe Institute (A. F. Ioffe RAS). The ultimate goal of the work is the development of technology and experimental samples of solar cells based on light-sensitive polymers and polymer–inorganic nanoparticle composites. Due to the unique set of properties characteristic of optically active conjugated polymers and their composites with inorganic nanoparticles, such structures will effectively absorb light in various spectral ranges, from ultraviolet to infrared [21,22,23,24,25,26].

Due to the fact that such polymers are soluble in common organic solvents, it becomes possible to deposit composites on substrates using inkjet and cold stamping technologies. This makes it possible to integrate the technology of composite solar cells into the technology of flexible printed electronics. The development of solar cells based on multilayer composite structures (polymer–inorganic nanoparticles) will increase the efficiency of such devices and increase the operating time of hybrid solar cells. To date, the efficiency of energy conversion in polymer tandem solar cells obtained in laboratory conditions reaches ~9%, which is already approaching the commercially competitive values of 10–11% [20].

## 3. Classification of Solar Panel Types

Solar panels are divided into three large families [27]:

(1) Thin-film solar panels consist of stretched films that can be easily installed in any convenient place. They are not afraid of dust and can work even in adverse conditions. In cloudy weather, their effectiveness is reduced by 20%. They are inexpensive, but require a large area for installation.
(2) Monocrystalline batteries are made from a large number of individual cells, which are filled with silicone. Thanks to their waterproofing, they are effectively used in shipping. Monocrystalline batteries are relatively light in weight and compact in size. They are distinguished by flexibility, light weight, compactness, reliability, and durability. They are easy to install and dependent on direct sunlight. In this case, even light cloud cover can lead to a cessation of energy production.
(3) Polycrystalline solar panels contain cells composed of crystals pointed in different directions. This makes it possible to capture diffused light and be less dependent on direct illumination. They are successfully used to illuminate houses, office buildings, and even streets.

Below are the physical and technical characteristics that significantly determine the functional component of the solar battery, regardless of the material of manufacture:

- Mechanical: geometric parameters; weight; size; number of cells; type and width of connectors.
- Temperature: one of the key factors is the change in efficiency when the temperature rises (in extreme conditions) by a certain unit of magnitude (usually 1 degree).
- Electrical or current-voltage: (CVC) power; open-circuit voltage; rate of change in current strength at maximum load; efficiency of individual cells and the panel as a whole.
- Functional: ease of use; performance; maintenance.
- High quality: service life; degree of degradation of cells; efficiency; versatility; environmental friendliness.

At the moment, eight main technologies can be noted in the production of highly efficient solar panels [28]:

- PERC (Passivated Emitter Rear Cell): dielectric layer on the reverse side of the cell.
- Bifacial: bilateral.
- Multi busbar: multiline.
- Split panels: half.
- Dual glass: frameless, with double glass.
- Shingled cells: inseparable elements.
- IBC (interdigitated back contact cells): interlaced contacts at the back of the cell.
- HJT (heterojunction cells): heterostructural cells.

**Figure 1:**
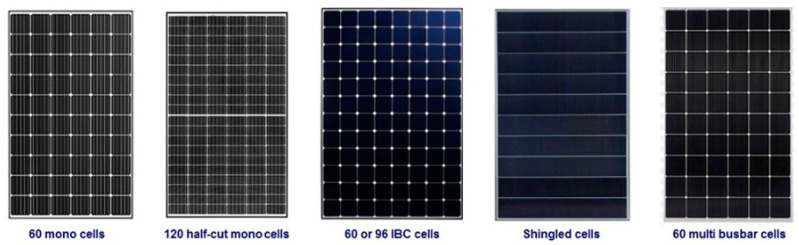
Different types of solar photovoltaic technology.

Five main types of solar panels using the latest solar photovoltaic technology in 2020 are presented in Figure 1:

Single-crystal silicon is a classic photovoltaic material; however, the production of structures based on it is a technologically complex and expensive process. Therefore, in recent years, more and more attention has been paid to materials such as amorphous silicon (a-Si:H), gallium arsenide, and polycrystalline semiconductors [28,29].

Direct conversion of solar radiation into electrical energy is carried out by solar photovoltaic cells (batteries, installations). The most widespread are solar photovoltaic installations (SPVI) based on three types of silicon: single-crystal, polycrystalline, and amorphous. In industrial production, there are SPVIs with the following efficiency [2]:

(1) Single-crystal: 15–16% (up to 24% on prototypes).
(2) Polycrystalline: 12–13% (up to 16% on prototypes).
(3) Amorphous: 8–10% (up to 14% on prototypes).

To develop cheap, high-efficiency Si-based solar cells using plasma-chemical technologies, it is necessary to use cheap multicrystalline substrates. It is promising to use thin multicrystalline Si layers deposited on mechanically strong substrates (glass, ceramics), since a silicon thickness of about 10 μm is sufficient for efficient operation of the SC. It is possible to create tandem heterojunction thin-film HIT SCs with a photoconversion efficiency higher than in the best samples of single-junction HIT structures (>25%). High Si-based efficiencies are likely to be obtained with triple or quad semiconductor junction technology [30].

HJT solar cells use a conventional crystalline silicon core with additional thin-film layers of amorphous silicon on either side of the cell, forming a so-called heterojunction. Unlike conventional P-N junction cells, multilayer heterojunction cells can greatly improve efficiency. Up to 26.5% efficiency has been achieved in laboratory tests when combined with IBC technology [28].

An especially promising technology involves the utilization of crystalline silicon thin films on glass substrates. This innovative approach capitalizes on the benefits of crystalline silicon as a solar cell material—its abundance, non-toxic nature, high efficiency, and long-term stability—while also harnessing the cost-effective advantages inherent in employing a thin-film methodology [31,32].

Another intriguing facet of thin-film solar cells is their capability to be deposited onto various materials, including flexible substrates like PET. This characteristic introduces novel opportunities for diverse applications [33].

As of December 2014, the pinnacle achievement in solar cell efficiency stood at 46%, attained through the utilization of multi-junction concentrator solar cells. This remarkable accomplishment was the result of collaborative efforts between Soitec and CEA-Leti in France, in conjunction with Fraunhofer ISE in Germany [34].

High-tech production allows manufacturers to produce products with amazing plasticity, with a light and thin module. To give thinness and lightness to flexible film solar cells, polymer sputtering with aluminum conductors is used. The panels can be rolled up and folded into a compact tube without damaging anything. The three basic components of these panels are flexibility, thinness, and lightness.

The solar panel, called eArche, created with the participation of Australian company Energus, is a super-flexible, ultra-thin solar array that can be placed on building facades, bus roofs, awnings, or other surfaces. According to project leader Dr Zhengrong Shi, the innovative panel has virtually unlimited potential, with 40 kW systems already in operation at three locations across Australia. According to the manufacturer, eArche has a thickness of 5–6 mm and weighs only about two tons per 100 kW, while conventional solar roof systems weigh about eight tons per 100 kW and cost about the same. The flexible photovoltaic panel can be custom-made to suit the individual sizes of the roofs and walls of buildings. It is also possible to use the technology for land vehicles, yachts, vending machines, and more [35].

## 4. Classification of Photovoltaic Materials and Manufacture Technologies

Industrial solar panels can be classified either by design features (standard design of a rigid solar battery, rigid and flexible panels made using various types of semiconductors) or by the type of working photovoltaic layer. In terms of the latter, there are the following classifications:

(1) SILICON-BASED

Crystalline silicon modules are characterized by two voltage ranges: 40–45 V for open circuit voltage and 32–38 V at the maximum power point (for modules in systems with an operating voltage of 24 V), as well as 20–25 V for open circuit voltage and 15–17 V at the point of maximum power (for modules in systems with an operating voltage of 12 V). Typical efficiency values for modules based on multicrystalline silicon lie in the range of 13–17% [36], and for monocrystalline in the range of 16–19% [37,38].

  - Monocrystalline (semi-flexible modifications)

Single-crystal silicon SCs (c-Si-SCs) are made from wafers 300 µm thick by doping them, creating ohmic contacts (solid rear and grating front), and texturing to impart antireflection properties. There are several types of construction of single-crystal and thin-film solar cells (SCs), which differ in the method of formation, structure, and arrangement of contacts [39,40].

  - Polycrystalline

Polycrystalline silicon solar cells generally perform better than some other types of solar cells at high temperatures. This makes them well suited for regions with high levels of sunlight and elevated temperatures.

  - Amorphous

It is predicted that by 2023–2025 amorphous cells will take a leading position in the development of solar energy.

The third generation includes solar cells with which it is possible to overcome the known efficiency limit of the Shockley–Kweiser photoelectric conversion [41].

  - CZTS—sulfide of copper, zinc, tin, and derivatives of CZTSe and CZTSSe

An emerging material for use in photovoltaic solar cells, CZTS silicon-based photovoltaic layers offer the advantages of abundance, non-toxicity, and a direct bandgap, making them an attractive candidate for solar cell applications. However, challenges related to efficiency, manufacturing scalability, and material quality need to be addressed to fully harness their potential in commercial solar panel technology. A transparent conductive layer, often made of materials like zinc oxide (ZnO) or indium tin oxide (ITO), is deposited on top of the CZTS absorber layer. This layer allows light to pass through and contributes to electrical conduction.

  - DSSC, DSC, DISC: dye-sensitized solar cell (“Grätzel cell”)

For the manufacture of silicon-free film photovoltaic cells, the main alloys used are: cadmium telluride (CdTe), indium-copper selenide (CIS), and indium-copper-gallium selenide (CIGS). CdTe stands out for its rapidly growing efficiency, stability, and durability. The use of these alloys has an efficiency of more than 30%. They are more expensive than their silicon counterparts, but thanks to their exceptional characteristics, they have occupied a high-tech niche for new generations of modules. It is important to note that the problem with the disposal of rare earth poisonous elements cadmium (toxic metal), rather expensive ones such as tellurium (comparable to the rarity of platinum), gallium, indium, germanium, and others, increases the technology required for their production. Possible combinations demonstrating the photoelectric effect: Cu, Ga, Ag, Se, Te, Au–Al, and In–S.

(2) TELLURIUM-CADMIUM BASED

Panels made of CdTe are actively used in the cladding of buildings, where extreme surface heating reaches 70–80 degrees.

Cadmium telluride (CdTe) is another promising material for photovoltaics. It has an almost ideal band gap and a very high radiation absorption capacity. CdTe films are quite cheap to manufacture. In addition, it is technologically easy to obtain a variety of CdTe alloys with Zn, Hg, and other elements used to create layers with the desired properties. Like CuInSe2, the best CdTe-based cells include a heterojunction with CdS as the window layer. Tin oxide is used as a transparent contact and antireflection coating. A serious problem with using CdTe is the high resistance of the p-CdTe layer, which leads to large internal losses. However, it is solved by a p-i-n structure with a CdTe/ZnTe heterojunction. CdTe films have a high mobility of charge carriers, and solar cells based on them have high efficiency values (from 10 to 16%) [42]. Table 1 contains the main characteristics of solar cells based on CdTe.

CdTe SCs are quite promising, with ample opportunities for improving and optimizing the production technology and, consequently, for reducing the cost [47,48,49]. This favorably distinguishes them from solar cells based on a-Si, c-Si, and A IIIB V semiconductors. However, in the production of CdTe SCs, Cd and Te, which are rare earth elements with strong toxic properties, are involved, which to some extent delays the widespread adoption of CdTe solar cells. In the near future, the world community plans to completely abandon the use of Cd in industrial production due to its high toxicity and problems with the disposal of Cd and its compounds. Therefore, when CdTe is chosen as an absorbing material for photovoltaic light conversion, there immediately arises the problem of further utilization of solar cells that have served their time. The latter leads to an increase in the cost of CdTe solar cells and limits their widespread use for light conversion [49].

Tiwari and collaborators [50] have announced the creation of a flexible CdTe solar cell with a conversion efficiency of 8.6%. In addition, flexible solar cells based on organic semiconductors and the so-called dye-sensitized solar cells are currently promising. Batteries of this type can be printed on polymer foil [51].

(3) BASED ON INDIUM-COPPER-GALLIUM SELENIDE

The CIS copper-indium selenide film is characterized by high efficiency and the ability to convert up to 20% of solar energy.

Elements based on a composite mixture of indium-copper-gallium selenide Cu(InGa)Se2 have the highest absorption coefficient of semiconductors presented in photovoltaics. The film thickness, which does not exceed hundreds of nanometers, provides high conversion efficiency, being the “champion” in terms of durability and efficiency.

Flexible copper-indium-gallium diselenide (CIGS) solar cells formed on stainless steel foil achieve 17.4% conversion efficiency [52,53].

At the same time, for CIGS-based elements formed on PI substrates that withstand relatively low deposition temperatures, the conversion coefficient decreases to 11% [53,54,55].

(4) BASED ON GALLIUM ARSENIDE

The best materials to combine with silicon in terms of efficiency are Group III–V semiconductors, especially GaAs, with which laboratory samples of elements have shown an efficiency of more than 32%, or perovskites based on metal halide, for which laboratory samples have shown an efficiency of 29.8%, according to Henry Snaite, Global Energy expert Professor of Physics at the Clarendon Laboratory at the University of Oxford. Group III–V semiconductors are still produced using very expensive and slow molecular beam epitaxy, which makes them prohibitively expensive. In contrast, metal halide perovskites can be produced very quickly at a low temperature using conventional thin film manufacturing processes, making them very attractive from an economic point of view [56].

Gallium arsenide (GaAs) solar cells are a type of high-efficiency photovoltaic technology that utilizes a semiconductor material called gallium arsenide as the absorbing layer to convert sunlight directly into electricity. GaAs solar cells are known for their excellent performance in terms of efficiency, especially in comparison to traditional silicon solar cells. GaAs cells are rapidly gaining popularity nowadays and hundreds of research papers featuring this type of solar cell are published every year [57,58,59,60,61,62].

Gallium arsenide GaAs and indium phosphide InP cells maintain efficiency at extreme temperatures of several hundred degrees Celsius. Elements (on the order of 2–3 microns) based on GaAs demonstrate conversion efficiency up to 44%, acting as an ideal semiconductor for photovoltaics. Methods for obtaining a multilayer structure based on GaAs with the inclusion of other materials made it possible to significantly increase the efficiency of using the invisible parts of the spectrum.

Currently, two- and three-layer photocells are being investigated, which allow one to study a large part of the solar spectrum along the wavelength of solar radiation. For a two-layer photocell on prototypes, an efficiency of 30% was obtained, and for a three-layer photocell, up to 40% was obtained [2].

(5) COMBINED AND MULTILAYER

Multilayer (multi-cascade) design increases the efficiency of flexible panels. The hybrid solar battery (PVT), utilizing the excess heat from photovoltaic cells, has opened up prospects for the generation of two energies: thermal and electrical. This symbiosis made it possible to halve the area required for installing thermal collector systems, thereby synchronizing the simultaneous operation of solar collectors and photovoltaic cells.

An innovative solution to achieving the efficiency of thin-film solar cells largely depends on the selected semiconductor and growing technology. Representatives of the third generation are thin-film flexible solar panels based on safe natural minerals, organics, and on the properties of physical quantum dots.

An alternative to the creation of solar cells is the manufacture of single-junction elements based on multilayer nanoheteroepitaxial structures (NHES) with quantum dots (QDs). In this case, the element design is greatly simplified, since such a solar cell is single-stage [63]. For their manufacture, two semiconductor materials are used: one is wide-gap (matrix), the other is narrow-gap (for the manufacture of QDs) [18].

(6) POLYMERIC

Polymer-based photovoltaic layers are often thin and flexible, allowing them to be integrated into various surfaces, including curved or flexible substrates. This property enables applications in wearable electronics, building-integrated photovoltaics, and other unconventional settings. They can be manufactured using solution-based processes, such as roll-to-roll printing or spray coating. These techniques are potentially more cost-effective than the high-temperature and vacuum-based processes used in traditional silicon solar cell production. The absorption characteristics of polymer-based photovoltaic materials can be tailored by adjusting their chemical structure. This tunability allows for the customization of the cell’s absorption spectrum to better match the solar spectrum. While polymer solar cells have made significant improvements in efficiency over the years, they generally have lower conversion efficiencies compared to silicon solar cells. However, their lower cost and ability to cover larger areas may offset the lower efficiency in some applications. Overall, polymer-based photovoltaic layers offer versatility, potential for low-cost manufacturing, and the ability to be integrated into a wide range of applications. While they may not yet match the efficiency of traditional silicon solar cells, their unique properties make them promising candidates for specific applications and for driving innovation in the solar energy field.

(7) ORGANIC

An example of some of the successes in the field of organic solar cells is the introduction made by Brabec and collaborators of a bulk heterojunction battery with a conversion efficiency of 5% [64]. The main problems in the creation of organic solar cells and solar cells that use dyes to increase sensitivity are the need to increase the absorption of visible light by the working layer [51] and to ensure photochemical stability of dyes [65].

In this case, the most important problem is the durability of the use of organic solar panels [66].

Table 2 summarizes and provides concise explanations of the most important parameters of photovoltaic materials.

Currently, there are several manufacturing technologies for photovoltaic materials that come with their set of advantages and shortcomings. Quantum dot (QD), quantum well (QW), and quantum superlattice solar cells are advanced photovoltaic technologies that leverage quantum mechanics principles to enhance the efficiency of solar energy conversion. Here is an overview of each type and their advantages:

Quantum Dot Solar Cells (QDSC)

Quantum dots are nanometer-sized semiconductor particles that exhibit unique electronic and optical properties due to quantum confinement effects. In QD solar cells, quantum dots are integrated into the solar cell structure to enhance light absorption and charge carrier generation. The following advantages are usually attributed to QDSC solar cells [67,68,69,70,71,72]:

  - Tunable bandgap: quantum dots’ size can be engineered to control their bandgap, allowing for absorption of a wider range of solar wavelengths and improved light absorption.
  - Multiple exciton generation (MEG): quantum dots can exhibit MEG, a phenomenon where multiple electron–hole pairs (excitons) are generated from a single photon. This increases the photocurrent and efficiency.
  - Reduced thermalization losses: quantum dots can reduce thermalization losses by rapidly transferring excitons to interfaces before they lose energy as heat.
  - Compatibility: quantum dots can be integrated into various solar cell architectures, including thin films and traditional silicon-based devices.

Quantum Well Solar Cells (QWSC)

Quantum wells are thin semiconductor layers that confine charge carriers to one dimension, leading to quantized energy levels. QW solar cells use these confined energy levels to enhance charge carrier separation and collection. Their attractiveness is explained by the following points [73,74,75]:

  - Enhanced carrier separation: quantum wells promote efficient charge separation due to the confined energy levels, reducing recombination losses.
  - Improved current generation: quantum wells facilitate the generation of multiple electron–hole pairs, similar to quantum dots, increasing the photocurrent.
  - Wide wavelength coverage: quantum wells can be designed to absorb specific wavelengths of light, enhancing the effective use of the solar spectrum.

Quantum Superlattice Solar Cells

Quantum superlattices are periodic arrangements of alternating thin layers with different bandgaps. They combine the advantages of both quantum wells and quantum dots, allowing for enhanced charge separation and light absorption. They particularly excel in the following aspects [76,77]:

  - Combined benefits: quantum superlattices leverage the advantages of quantum wells and quantum dots to achieve enhanced light absorption, charge carrier separation, and efficient collection.
  - Wavelength control: by engineering the thickness and composition of layers, the absorption can be fine-tuned for optimal solar spectrum coverage.

Overall Advantages of Quantum-Based Solar Cells

  - Enhanced efficiency: quantum confinement effects and the ability to generate multiple excitons from a single photon contribute to higher conversion efficiencies.
  - Tailored absorption: quantum-based solar cells can be designed to efficiently absorb specific portions of the solar spectrum.
  - Reduced thermalization losses: the rapid separation of excitons in quantum-based structures reduces thermalization losses.
  - Potential for low-cost fabrication: quantum dots and wells can be integrated into existing solar cell technologies, potentially allowing for low-cost fabrication methods.
  - Versatility: quantum-based structures can be combined with other advanced materials and technologies to further improve solar cell performance.
  - While these quantum-based solar cell technologies offer intriguing advantages, they also present challenges related to material synthesis, stability, and scalability. Ongoing research aims to address these challenges and unlock the full potential of quantum-based approaches for more efficient and sustainable solar energy conversion.

TOPCon is another innovative technology for solar cell manufacture that is designed to maximize efficiency and minimize energy losses. In these advanced solar cells, a thin-tunnel oxide layer is strategically placed between the silicon wafer and the contact layer. This tunnel oxide layer serves a dual purpose: it efficiently transports charge carriers while simultaneously reducing recombination losses at the surface. This innovative design enables improved charge carrier collection and enhanced passivation of defects, resulting in higher efficiency and overall performance [78,79].

The key feature of TOPCon solar cells is their ability to achieve low contact resistivity, meaning that the electrical contact between the silicon and the metal is highly efficient, allowing for better charge carrier extraction. The passivation properties of the tunnel oxide layer also reduce the risk of charge carrier recombination at the surface, thereby increasing the cell’s overall conversion efficiency. Additionally, TOPCon solar cells are compatible with various manufacturing techniques, making them a viable option for large-scale production.

The unique combination of tunnel oxide layer technology and effective passivation strategies in TOPCon solar cells has led to remarkable advancements in the efficiency of silicon solar cells. With their promise of higher efficiency, improved temperature stability, and compatibility with existing production processes, TOPCon solar cells hold significant potential for driving the evolution of solar energy technology and contributing to the transition to cleaner and more sustainable energy sources.

## 5. Perovskite and Kesterite Solar Cells

Calcium titanate CaTiO_3_ (perovskite) is one of the more abundant minerals on earth. In almost all respects, perovskite outperforms competitors, including in the average cost of electricity over the entire life of a solar cell from a given material (levelised cost of energy, LCOE). Difficulties are possible only with the disposal of obsolete panels due to the toxicity of perovskite compounds. [Source: Group for Molecular Engineering of Functional Materials (GMF), Switzerland]

The main advantage of perovskites is that they can be made from common metals and industrial chemicals rather than the expensive raw materials used in other silicon-based solar cell substitutes. In addition, the application of photosensitive elements based on perovskites directly onto glass (or other materials) is much cheaper than methods for obtaining thin-film elements [80]. This allows manufacturers to set up a large mass production that does not require huge expenditures of resources. Moreover, perovskites can be applied to flexible structures such as plastics and fabrics, which opens up great opportunities for their application. Another important advantage of perovskites is their stability. Even under continuous lighting conditions, the current conversion is reduced by only 10% of the original [81]. Experts speculate that in the next ten years, the efficiency of solar cells based on perovskites will reach 50% [82].

However, working with perovskite is not easy. Once applied to the film, perovskite crystallizes very quickly, making it difficult to create an even layer over a large area. This is the main task when creating a solar cell: to achieve as much surface area as possible while maintaining high energy conversion efficiency. In June 2018, Toshiba produced the world’s largest perovskite thin-film solar cell with the world’s highest energy conversion efficiency [83].

Regarding the efficiency of solar energy converters based on perovskite (organo-inorganic type), it is worth noting that in 2009 the efficiency of the first perovskite panels was only 3.5% and now, in 2020, the efficiency has increased to 22.7% (in laboratory conditions). Of course, they are inferior in terms of efficiency to traditional silicon solar cells, in which it is up to 30%, however, this indicator has not changed over the past 15 years [84].

Scientists have proposed a unique method for obtaining perovskite solar cells of unlimited area and have created solar cells with an efficiency of more than 17%. The results of the work are published in Nature Nanotechnology. According to a new method, a polyiodide melt is formed in situ directly on the surface of lead metal [85].

Polyvinylidene fluoride has made perovskite solar cells more stable and more efficient. During synthesis, this additive helps perovskite to crystallize faster, and during operation it acts as a kind of shock absorber, reducing friction between adjacent crystallites. The results of the study were published in the journal Science [86].

Fluctuations in daily temperatures lead to phase shifts and lattice distortions in halide perovskites, posing challenges to their stability in solar cell applications. By leveraging the structured dipolar arrangement of β-poly(1,1-difluoroethylene) it is possible to achieve stabilization of the perovskite black phase and enhancement of solar cell performance through controlled crystallization and energy alignment within the perovskite film. The research yielded p-i-n perovskite solar cells with an unprecedented power conversion efficiency of 24.6% across an 18 square millimeter area, and 23.1% across a 1 square centimeter area. These cells maintained 96% and 88% efficiency after 1000 h of continuous one-sun maximum power point tracking at temperatures of 25 °C and 75 °C, respectively. Furthermore, rigorous thermal cycling between −60 °C and +80 °C exhibited no signs of fatigue in the devices, underscoring the positive influence of the organized dipolar structure on the long-term stability and performance of perovskite solar cells [86].

One of the main advantages of perovskite solar cells is their ease of manufacture. Unlike silicon cells, which require silicon obtained from a multi-step purification process, perovskite cells can be made using the simplest laboratory equipment [87,88]. Chinese and American materials scientists led by Huan Ping Zhou from Peking University proposed to obtain perovskite layers using hot liquid firing (liquid medium annealing, LMA). The authors hypothesized that immersion in a hot liquid would help heat the substrates faster and keep them free of contaminants.

Perovskite-based solar cells have gained popularity due to their economic availability and a wide range of applications (industry, agriculture, everyday life, and even clothing) and act as an alternative to silicon.

Kesterite solar cells are a type of thin-film photovoltaic technology that hold the potential to harness solar energy through innovative semiconductor materials. These solar cells are based on a compound semiconductor material known as kesterite, with the chemical formula Cu_2_ZnSn(S, Se)_4_. The kesterite structure is composed of copper (Cu), zinc (Zn), tin (Sn), and sulfur (S) or selenium (Se) atoms arranged in a crystal lattice.

Kesterite solar cells are part of the broader family of thin-film solar cells, which are known for their potential to be lightweight, flexible, and relatively cost-effective due to reduced material usage in comparison to traditional silicon solar cells.

Key features and aspects of kesterite solar cells include [89,90,91,92]:

- Abundance of elements: kesterite solar cells are advantageous because they use earth-abundant and non-toxic elements, such as copper and zinc, in contrast to some other thin-film technologies that might rely on rarer or more expensive elements.
- Tunable bandgap: the bandgap of kesterite materials can be engineered by adjusting the composition of sulfur and selenium. This tunability allows the solar cells to absorb different portions of the solar spectrum and enhances their potential for efficient energy conversion.
- Multinary composition: kesterite solar cells are part of the family of chalcogenide-based photovoltaic materials, which includes various combinations of elements from Group I and Group II of the periodic table. These multinary compounds offer a degree of flexibility in bandgap engineering.
- Efficiency potential: kesterite solar cells have shown potential to achieve moderate energy conversion efficiencies, with records continuing to improve as research advances.
- Processing techniques: kesterite solar cells can be manufactured using techniques like co-evaporation or solution-based methods. These methods offer possibilities for cost-effective and large-scale production.
- Challenges: despite their promise, kesterite solar cells face challenges related to achieving high efficiency, ensuring material stability, minimizing defects, and addressing issues of grain boundaries and non-radiative recombination.

Researchers and industries are actively exploring ways to optimize the efficiency, stability, and manufacturability of kesterite solar cells to make them a viable and competitive option for harnessing solar energy. While still a developing technology, kesterite solar cells represent an interesting avenue for advancing thin-film photovoltaics and expanding the options available for renewable energy generation.

A comparison of perovskite and kesterite materials across different parameters is given below [93,94,95,96,97,98,99,100,101]:

- Carrier recombination:

Kesterite solar cells tend to have higher levels of recombination due to defects and grain boundaries in the material. These defects can trap charge carriers and lead to non-radiative recombination losses. Perovskite solar cells also face recombination challenges, especially in the form of interface recombination at the perovskite/transport layer interfaces. However, advances in passivation techniques have helped mitigate recombination losses to some extent.

- Carrier lifetime:

The carrier lifetime in kesterite solar cells can be relatively short due to the presence of defect states. This can limit the time charge carriers spend within the material before recombining. Perovskite solar cells have demonstrated relatively long carrier lifetimes, especially in high-quality perovskite films. This longer lifetime enhances charge carrier extraction and contributes to higher efficiency.

- Voc bottleneck:

Kesterite solar cells often face challenges in achieving high open-circuit voltage (Voc) due to recombination losses and the presence of defect states. Perovskite solar cells have made impressive progress in improving Voc through passivation techniques and bandgap engineering. However, some perovskite materials still face limitations in achieving higher Voc due to defects and material instability.

- Effective use of solar spectrum:

Kesterite solar cells have the advantage of tunable bandgaps, which can be optimized to better match specific portions of the solar spectrum. This can lead to efficient utilization of sunlight. Perovskite materials can also be engineered to achieve desirable bandgaps, enabling effective absorption of a broad range of solar wavelengths.

- Stability and reliability:

Kesterite solar cells have faced challenges related to long-term stability, particularly in the presence of moisture and oxygen. This instability can lead to efficiency degradation over time. Perovskite solar cells have made significant progress in stability but still encounter issues related to moisture, temperature, and other environmental factors. Ongoing research aims to improve the long-term reliability of perovskite devices.

Both kesterite and perovskite solar cells are promising emerging technologies that offer the potential for low-cost, high-efficiency solar energy conversion. They both require careful engineering to address recombination, stability, and efficiency challenges. Both technologies benefit from advances in passivation techniques, improved material quality, and innovative device architectures to enhance their performance.

Kesterite solar cells are based on compounds containing abundant elements like copper, zinc, tin, and sulfur, while perovskite solar cells use organometal halide compounds. Perovskites have achieved higher efficiency records in a relatively short time but still face stability challenges. Kesterite solar cells have a longer history but tend to lag in terms of efficiency and stability compared to some advanced perovskite materials.

In summary, while both kesterite and perovskite solar cells show promise, they each have unique strengths and challenges. Ongoing research and development efforts are critical for addressing their limitations and unlocking their full potential for efficient, stable, and cost-effective solar energy conversion.

## 6. Current Advances in the Flexible Solar Panel Industry

To date, the use of solar panels in light industry products is not widespread enough. For example, the world-famous company Tommy Hilfiger launched a “pilot” project for the use of solar panels in clothing and developed jackets with solar panels built into the design of the product. The manufacturer did not hide the batteries but made them part of the design. The built-in 6000 mAh battery is capable of accumulating enough energy for several smartphone charges [102].

One of the promising areas is the introduction of elements that accumulate solar energy into the structure of a garment in order to use this energy for domestic purposes, for example, to charge gadgets. This idea is promising in the use of departmental clothing, including equipment for the police, the army, and other special-purpose clothing [103].

Scientists from Australian National University (ANU) successfully demonstrated a straightforward technique to enhance the structural integrity of low-to-moderate bandgap perovskites devoid of methylammonium (MA) by introducing 4M-PEACl into the perovskite precursor [104]. Despite the fact that 4M-PEACl did not integrate within the three-dimensional perovskite lattice, this method effectively facilitated the transformation into the α-FAPbI3 perovskite phase. This transformation notably bolstered crystallinity while concurrently mitigating defects such as twin grains within the perovskite films. The presence of quasi-2D perovskite components along the grain boundaries of the 3D perovskite structure contributed to the amelioration of defect passivation and the enhancement of internal carrier transportation properties. Moreover, the incorporation of 4M-PEACl induced significant alterations in the surface chemistry of the perovskite films, leading to a more favorable electronic structure for hole extraction. The consequential outcome was a marked enhancement in the efficiency and light stability of perovskite solar cells. Remarkably, the MA-free perovskite solar cells attained a consistent efficiency of 23.7% (24.2% in reverse scan, 23.0% in forward scan), with over 93% of the initial performance retained even after 1000 h of continuous illumination.

An innovative proposal was made by a group of scientists from RIKEN–Toray Industries Inc from Tokyo: elastic ultra-thin (3 μm) solar cells. New batteries recover their shape after being stretched or compressed, are securely attached to fabric or another surface with hot-melt adhesive, and withstand temperatures up to 100 degrees Celsius [105].

The developers of the RIKEN Institute and the University of Tokyo have created an ultra-thin flexible photocell that does not exclude direct contact with water and is not afraid of tensile loads. By integrating flexible photovoltaic systems into textiles, materials with the widest functionality for creating “smart” clothes are formed.

Giving products an additional functional load is becoming a sustainable trend in the design and production of clothing [103].

“Wearable” technology is represented by intelligent, technological clothing, footwear, and headwear with built-in electronics with the ability to connect mobile devices. The use of flexible batteries in textile production to create the so-called “photo curtains” is another example. Flexible solar modules while generating energy at the same time protect the room from excessive penetration of sunlight, which ensures a comfortable indoor climate.

A technique for designing special-purpose headgear in three-dimensional graphic CAD (computer-aided design) was proposed, coupled with planar design and modeling modules “Eleandr–konstruktor” [106] and “Eleandr-KM” [106]. The result of the experimental study was a developed constructive and technological solution for a special-purpose headgear: a cap with removable batteries [107].

In the case of solar cells integrated into clothing, it is necessary to use substrates thin enough so that the solar cells do not significantly change the “flexibility” of the material onto which they are placed. Note that it is also necessary to carry out work on the creation of a thin and strong material for encapsulating solar cells. The authors note that, taking into account the possible location of a solar battery with an area of 1000 cm^2^ on the clothes of an adult, flexible solar cells integrated into clothes can provide the necessary energy consumption [108].

Although it is too early to talk about specific economic indicators of the use of perovskite, since the widespread practical use of this material in solar cells is predicted after 2025, the “Russian mineral” has the prerequisites for a great and successful future. According to experts from the US National Renewable Energy Laboratory (NREL), the production of perovskite panels will be ten times cheaper than that of their silicon counterparts. There are two more advantages of perovskite solar cells: flexibility and transparency. The application of perovskite to a polyurethane substrate is already known about, the efficiency of which in absorbing the sun has reached 5.72% [83].

Scientists from the Moscow Institute of Steel and Alloys (MISiS) and St. Petersburg University of Information Technologies in Mechanics and Optics have developed a perovskite-based solar cell that can simultaneously operate as a battery and as an LED and is based on perovskite halide [83].

Along with traditional mono- and polycrystalline solar panels on a rigid frame, flexible thin-film panels are widely used. The technological process of creating thin-film solar cells formed on flexible substrates is relatively simple, and minimal energy consumption significantly reduces the cost of manufacturing “flexible” solar cells.

Flexible solar panels are quite widely represented on the market, taking into account their indicative characteristics:

- Current strength;
- High efficiency;
- Additional features (models with suction cups, with fastening on a backpack);
- Reliable functioning;
- Efficient work.

The production of a-Si solar cells is already at a fairly high technological level. As the main technological process, thin-film technology of plasma-assisted chemical vapor deposition from silicon- and germanium-containing mixtures (SiH_4_, Si_2_H_6_, GeH_4_) is used [42].

Photovoltaic film in micromorphic (polymorphic) panels forms a multilayer a-Si cake with distinct properties in each of the layers.

The main characteristics of some solar cells based on silicon (single-crystalline c-Si, microcrystalline mc-Si, thin-film tf-Si, and amorphous a-Si) are presented in Table 3.

It should be noted that optimized solar cells based on single-crystal silicon are superior in their parameters to cells based on α-Si in the range of illumination intensities from medium to low levels due to higher conversion efficiency. At the same time, at a very low illumination level of less than 500 lux, the α-Si:H element outperforms the c-Si element [109,110]. A substantial portion of incident energy in photovoltaic solar energy conversion devices goes to waste, primarily due to the necessity of having a gap within the electronic states’ continuum of the light-absorbing material, which functions as an absorption threshold. When photon energies exceed this bandgap, any excess energy is converted into heat through the thermalization of excited charge carriers. Meanwhile, light with energies below the bandgap remains unutilized [41,111,112]. In materials with low bandgaps like germanium or crystalline silicon (c-Si), power conversion losses are mainly attributed to thermalization. However, in materials with bandgaps larger than 1.3 eV—common in thin-film photovoltaic absorbers and oxide-based electrode materials for photoelectrochemical energy storage—transmission of sub-bandgap light becomes the predominant factor [113,114]. This phenomenon even affects solar cells fabricated from c-Si, leading to a significant portion of the solar spectrum being lost through transmission [115].

The use of nanostructured amorphous silicon in combination with an additional layer made by conventional technology:

- Increased the conversion efficiency of the infrared part of the spectrum, while there were no losses in the absorption coefficient in the visible part;
- Allowed for the minimization of the overall thickness of the film;
- Reduced the rate of degradation by 20–25%.

Amorphous silicon solar cells with a p-i-n structure are now used in a wide variety of fields due to the possibility of their fabrication on metal foils, such as stainless steel, and polymer films provided with a metal coating. The use of such substrates is compatible with the technology of mass production of flexible solar cells. Therefore, elements of this type are among the most promising solar energy converters of the near future [116].

Active innovative development of the solar energy complex (development and implementation of advanced technologies for creating varieties of flexible solar panels) is accompanied by its spatial expansion, which is due to these positive aspects:

- Reliability (the product is protected from mechanical damage, moisture, and high temperatures, many designs are equipped with covers).
- Impact resistance (the panel remains intact when dropped from a height of many meters. The elements are relatively insensitive to overheating and are suitable for creating installations of any power).
- Thin and pliable structure (sufficiently large bending radius allows installation of buildings with a non-linear roof shape, dismantling of surfaces of any configuration, and versatility, i.e., the ability to fold and roll into a roll).
- Lightness (selenium, silicon, etc., are used to create semiconductors. Polymer sputtering and aluminum conductors lighten the panel by reducing the load on the supporting structures of structures).
- Environmental friendliness (materials can be recycled and reused. The source of energy is environmentally friendly, silent, and renewable. The daily influx of sunlight to the Earth is 120 thousand terawatts, which exceeds the need of earthly civilization for energy more than 20 thousand times).
- Availability and ease of operation (easy to install, simple equipment maintenance, do not require highly qualified personnel).
- Profitability (high overall energy production as power efficiency is maintained in cloudy weather), and a high level of optical absorption of photons increases the efficiency of batteries, simplifies production technology, reduces energy payback time for modules, has a favorable cost, and allows for energy supply to a country (providing autonomous control of heating, ventilation, and lighting, with an energy efficiency index from 14% to 30% and excellent maintainability as it is enough to cut out the damaged area, replace it with a new one and reconnect it to the circuit).
- Performance characteristics (wide range of applications, significant working life due to high ability to capture various spectra of solar radiation, high-quality operation in low light conditions, performance drop with significant temperature fluctuations is not critical, and a snug fit to the surface guarantees resistance to wind loads). Versatility and simplicity, i.e., the ability to increase power by adding new modules.
- Aesthetics. Modules can become part of the design idea, be part of the architectural decoration of houses, and play the role of home decoration.

Numerous photovoltaic energy conversion systems miss out on harnessing the solar spectrum’s lower-energy photons that lie below the bandgap. This is particularly evident in materials with high bandgaps like amorphous silicon, polythiophenes, and lead halide perovskites. A method of photochemical upconversion, employing triplet-triplet annihilation (TTA-UC) within organic molecules, presents a flexible solution for capturing and converting this untapped light into wavelengths beneficial for the respective device [115].

Research in this area has predominantly revolved around two distinct yet interrelated approaches [117,118]. Firstly, the incorporation of lanthanoid cations (primarily erbium and ytterbium) into a solid-state matrix has been explored [119]. These materials leverage their well-defined and long-lasting atomic states, enabling the absorption of multiple photons and subsequent upconverted luminescence. As rare-earth materials absorb within the infrared region of the solar spectrum, lanthanoid upconversion (L-UC) holds promise, particularly for enhancing c-Si solar cell performance [120,121,122].

Experiments with lanthanide boost converters have shown that the advantage of the boost converter layer is limited by the high light intensity required to achieve high conversion quantum efficiency. To address this limitation, boost-converting materials can be combined with quantum dots or plasmonic particles to improve conversion efficiency and improve the applicability of boost converters in commercial solar cells.

The defining directions in the development of various types of flexible solar panels are technologies in the study of fundamentally new materials, with characteristic indicators of reliability, durability of the service life, and compliance with the price and quality of the product.

The comparative cheapness of solar cells formed on flexible substrates compared to solar cells created on glass is associated primarily with the possibility of using the so-called roll-to-roll method in their production. In addition, the reduction in energy consumption when creating solar cells on flexible substrates compared to batteries formed on glass is associated with the use of plastic substrates as flexible substrates, which do not require significant energy costs during their formation. Therefore, despite the fact that in recent years the production of thin-film silicon solar cells formed on glass substrates with an area of up to ~6 m^2^ [123,124] has been significantly increased, there is a need to develop new approaches to the creation of solar cells that allow for increasing the productivity of technological lines by an order of magnitude while significantly reducing costs.

To compete with large-area photovoltaics formed on glass plates, the method of manufacturing photovoltaic modules based on “roll coating” must solve a number of problems related to substrate cost reduction, technological simplicity, and low-cost strong encapsulation. Taking into account the importance of all the above tasks associated with the production of “flexible” solar cells, the authors considered the problems associated with low-temperature deposition and the formation of a sequential structure of a solar cell [125].

The rapidly developing industry of the solar energy complex offers the most durable, productive, and powerful examples of flexible solar panels based on the improvement of traditional and the use of fundamentally new materials:

- Manufactured using silicon-free technology;
- Based on amorphous silicon;
- Based on nanoheteroepitaxial structures with quantum dots.

Interest in solar cells on flexible substrates is increasing from developers of autonomous systems of orbital stations and telecommunications satellites, as well as specialists working in the field of rural electrification in developing countries. Small aircraft, automobiles, and other consumers of electrical energy can obtain some of the energy they need from the ambient lighting of their exposed surfaces using flexible photovoltaic cells. Another application of “flexible” photovoltaics is the integration of small photovoltaic systems into clothing to power portable electronic devices [109,126,127].

In addition to the need to optimize the material and structure of the solar battery in the case of using flexible substrates, it is necessary to solve the problem associated with the series connection of individual solar cells in a photovoltaic module [128]. Monolithic serial connection of individual elements allows for an increase in the voltage of photovoltaic modules for low-cost photovoltaic systems [129].

In [130], the authors present the results of research on optimizing the low-temperature deposition of solar cells based on hydrogenated silicon films consisting of amorphous hydrogenated silicon, nanocrystalline hydrogenated silicon, and protocrystalline hydrogenated silicon (pc-Si:H).

The authors also investigated solar cells based on an optimized solar cell using pc-Si:H deposited on a flexible low-cost polymer substrate at substrate temperatures of 40 °C < T_d_ < 110 °C. The experiments performed showed that for a temperature T_d_ = 110 °C, in the case of using non-textured PET foil, the conversion efficiency was 9%. A solar module with an area of 4 × 10 cm^2^, based on a single-element α-Si pin structure formed on polyethylene naphthalate (PEN), demonstrated a conversion efficiency of 3% [131].

It is assumed that the optimal material for creating an absorbing layer of α-Si solar cells is undoped protocrystalline silicon [132], which has better electronic parameters than α-Si:H and nc-Si:H. When using low substrate temperatures Td < 100 °C, pc-Si:H is the only optimal material for creating a thin-film Si-based solar cell [133]. At the same time, at T_d_ > 200 °C, the parameters of the solar cell improve when moving from α-Si:H to pc-Si:H when forming a light-absorbing layer [134].

In [108], the author presents a review of available data on the properties of hydrogenated silicon films, conditions for obtaining protocrystalline silicon, and the parameters of solar cells based on their flexible substrates.

The use of flexible substrates (stainless steel foil or polymer foil) somewhat simplifies and reduces the cost of forming a solar cell, which is partly due to the possibility of using the “roll to roll” technique. At the same time, in order to form solar cells based on hydrogenated silicon films on flexible polymer substrates, it is necessary to refine the technology for producing solar cells based on this material at relatively low temperatures (<150 °C). The preferred material for forming the active layer in this case is protocrystalline silicon pc-Si:H, the technological conditions for the formation of which significantly depend on the ratio of gases in the hydrogen–silane mixture and the design of the reactor for depositing films of the structure. Currently, silicon solar cells have been obtained on non-textured PET foil at Td = 110 °C with a conversion efficiency of ≈5% [128].

The possibility of depositing films onto stainless steel and heat-resistant polymer substrates allows for the creation of flexible, portable solar modules and the carrying out of deposition over relatively large areas. The main problem of these modules based on amorphous silicon is low resources, due to the removal of hydrogen from the films under the influence of temperature and solar radiation (the Stebler–Vronsky effect) and the low efficiency of the module, which lies in the range of 4–7% [135].

For the first time, cylindrical solar cells were developed by the American company Solyndra, using copper, gallium, indium, and selenium. Cylindrical solar panels capture more light, showing high performance. The presence of a cylindrical shape means resistance to strong winds, with gusts up to 200 km/h. The first large roll-up flexible solar array was used in NASA’s Solar Array Flight Experiment (SAFE) program. The first foldable flexible solar panel was installed on the Communications Technology Satellite (CTS) [136].

**Figure 2:**
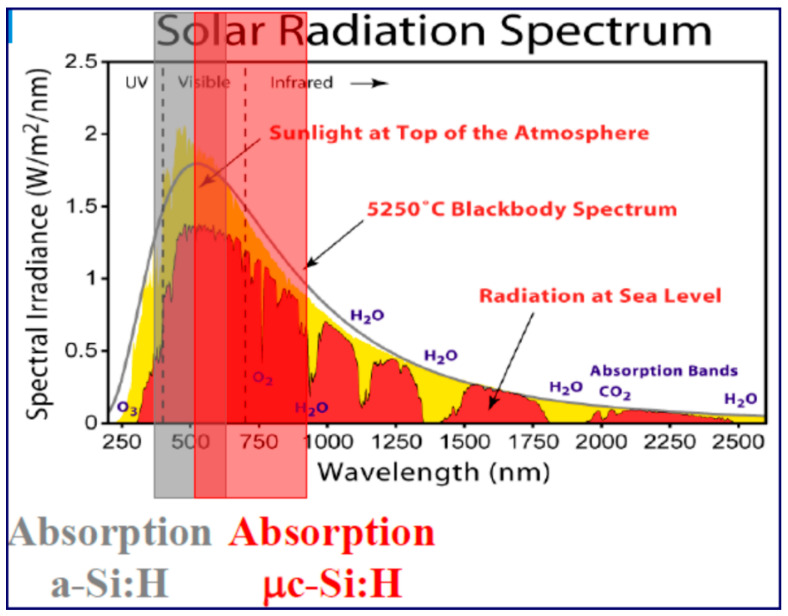
Areas of the solar radiation spectrum which are absorbed in the layers of an amorphous silicon (a-Si:H) and microcrystalline silicon (μc-Si:H) doped hydrogen cell [135,138].

Oerlikon Solar (Switzerland) and Applied Materials (USA) have developed similar technological processes for the production of solar cells and tandem-type modules: an amorphous silicon layer is supplemented with a layer of silicon microcrystallites [137]. The band structure of amorphous silicon is such that it allows the absorption of photons in a slightly different part of the spectrum than for crystalline modules (Figure 2). Therefore, the combination of layers of amorphous and microcrystalline silicon expands the module’s capabilities for converting reflected and scattered solar radiation. In addition, the dependence of these characteristics on ambient temperature for such modules is usually lower than for crystalline ones (Table 4) [138].

The main technologies for non-silicon solar cells are thin films based on cadmium telluride (CdTe) and composites based on copper, indium, gallium, and selenium (CIGS). The technology for preparing these films also allows scaling over sufficiently large areas and the use of flexible substrates. Plasma chemical or chemical vapor deposition is usually used [139], as well as magnetron sputtering [140] and liquid phase inkjet printing followed by heat treatment [141]. The efficiency of modules based on CdTe technology lies in the range of 8–11%, and CIGS in the range of 9–14%, which is already approaching the efficiency of multicrystalline PEMs [142]. The record holders for module efficiency are PEMs based on heterostructures of the A3B5 type (24–27%) [143], however, the high cost of the materials used makes it necessary to produce small-area solar cells from such modules [138].

The research [144] outlines techniques for achieving ultra-thin GaAs photovoltaics using interlayer adhesiveless transfer-printing of vertical-type devices onto metallic surfaces. These vertical-type GaAs photovoltaic devices utilize bottom electrodes to recycle reflected photons. Through systematic examinations involving four distinct solar microcell variations, it has been determined that these vertically oriented solar microcells, which are just a quarter of the thickness of equivalently designed lateral-type cells, can produce a comparable level of electrical power to their thicker counterparts. Both the empirical findings and the theoretical analysis presented in this study demonstrate the robustness of the ultra-thin vertical-type solar microcells even under extreme bending, making them well-suited for integration into the fabrication of flexible wearable electronics.

Lately, there has been a growing emphasis on foldable electronics technology within both academic and industrial research communities. This technology differs from flexible technology due to its requirement to endure substantial mechanical strains, particularly those resulting from an extremely tight bending radius of 0.5 mm. Achieving foldable devices demands transparent conductors with remarkable mechanical durability, necessitating their ultra-thin dimensions in the micrometer range and integration of the conductive material into a substrate.

In the study [145], a composite film of single-walled carbon nanotubes (CNTs) and polyimide (PI) is synthesized, boasting a mere 7 µm thickness. This composite film serves as a foldable transparent conductor for application in perovskite solar cells (PSCs). During the high-temperature curing process of the CNT-embedded PI conductor, the CNTs undergo stable and robust p-doping through the utilization of MoOx. This results in heightened conductivity and improved hole transportability. The incredibly thin foldable transparent conductor exhibits a sheet resistance of 82 Ω/sq.^−1^ and a transmittance of 80% at 700 nm. When incorporated into a foldable solar cell, it achieves a maximum power point tracking output of 15.2%.

Remarkably, these foldable solar cells can endure over 10,000 folding cycles at a 0.5 mm folding radius, showcasing an unprecedented level of mechanical resilience. Furthermore, these solar cells exhibit superior performance compared to other flexible solar cells based on carbon nanotube transparent electrodes, marking a significant advancement in the field.

Elevated criteria for foldable electronics, necessitating resilience under rigorous mechanical demands such as a bend radius of 0.5 mm, have been successfully addressed through the introduction of an ultra-thin matrix composed of MoOx-doped single-walled carbon nanotubes (SWNT) and polyimide (PI). The ultra-thin transparent conductor based on SWNT-PI displayed outstanding mechanical durability, boasting a fineness of 7 µm, a smooth surface morphology, and a high ratio of direct current (DC) to optical conductivity. Capitalizing on the mechanical robustness of SWNT, the SWNT-PI conductor also showcased exceptional adhesion and ultra-thin properties, making it an ideal candidate for applications in foldable electronics [145].

The authors of the work [146] fabricated ultra-thin flexible SCs based on InGaP/GaAs HES with a reverse layer sequence grown by MOCVD on a germanium substrate 100 mm in diameter. The area of ultra-thin flexible SCs was ~1 cm^2^.

Researchers from South Korea have achieved the development of solar cells with such slender dimensions that they can be contoured around diverse objects. To realize these ultra-thin solar cells, the scientists undertook the deposition of gallium arsenide semiconductor cells onto an incredibly thin and flexible substrate. Employing a temporary adhesive layer, the cells were affixed to the substrate’s surface, a process executed within a specialized chamber set at a temperature of 170 degrees Celsius. Through a procedure termed “cold welding,” the cells were effectively fused with the substrate. Subsequently, the adhesive layer was meticulously removed, resulting in a significant reduction in the thickness of the photovoltaic cell. However, the technology pioneered by the Korean researchers must still undergo an array of comprehensive assessments and evaluations before it becomes viable for widespread incorporation into the production of various items [144].

A team of researchers from Rice University, Houston Community College, and Brookhaven National Laboratory have developed flexible organic photovoltaic wafers that can be used to generate constant power. The study is published in the journal Chemistry of Materials. Organic solar cells are made up of carbon-based materials, including polymers, which store sunlight and convert it into electricity [147].

Scientists from Stanford University have created flexible solar panels that can be made using existing technologies, and any material, for example, an ordinary sticker, can be used as a substrate for them. The authors of the work tested the application of a flexible solar battery on various surfaces (glass, plastic, paper) and the device worked on all of them without loss of efficiency. The researchers point out that the production of such solar cells is not only easier and cheaper than traditional photovoltaic panels of the appropriate size, but also devoid of any waste. Silicon wafers can be reused [148].

Another team of scientists have developed solar panels as thick as spiderwebs. Specialists have created ultra-thin, flexible, and very efficient solar cells. The third part of the cell is the active elements that are engaged in the production of energy, and 2/3 of the cell is the polymer base [149].

The novel technique involves an inverted growth process for the solar cell. These layers incorporate high-energy materials featuring exceptionally high-quality crystals, particularly in the upper layers where the majority of power generation occurs. Not all layers conform strictly to a lattice pattern with uniform atomic spacing. Instead, the cell encompasses a comprehensive array of atomic spacing, facilitating enhanced sunlight absorption and utilization. The bulky and rigid germanium layer is eliminated, leading to a reduction in both the cost and weight of the cell by 94%. By overturning the conventional approach to cell fabrication, the outcome is an exceptionally lightweight and flexible cell that also achieves a groundbreaking level of solar energy conversion efficiency (40.8% under 326 suns’ concentration).

Experimental photovoltaic panels that diverge from traditional silicon can be fashioned using quantum heterostructures, such as carbon nanotubes or quantum dots, which are integrated into conductive polymers or mesoporous metal oxides. Furthermore, the application of thin films of these materials onto conventional silicon solar cells can heighten the efficiency of optical coupling into the silicon cell, leading to an overall enhancement in efficiency. By manipulating the dimensions of the quantum dots, the cells can be finely adjusted to absorb distinct wavelengths of light. Although the investigation is still in its preliminary stages, photovoltaics enhanced with quantum dots hold the potential to attain an energy conversion efficiency of up to 42%, attributed to the phenomenon of multiple exciton generation (MEG) [150].

An emerging field has arisen that employs networks of carbon nanotubes as transparent conductors in organic solar cells. These nanotube networks possess flexibility and can be applied to surfaces through various methods. Through specific treatments, nanotube films can achieve significant infrared transparency, potentially facilitating the development of effective low-bandgap solar cells. It is noteworthy that nanotube networks function as p-type conductors, which sets them apart from the conventional n-type transparent conductors. The introduction of a p-type transparent conductor could pave the way for novel cell designs that streamline the manufacturing process and enhance overall efficiency.

Efficiency and reliability determine the advantage of half-cut solar panels over their solid-cell counterparts, leading to savings in installation time and costs.

The ninth edition of ITRPV (International Technology Roadmap for Photovoltaic) predicts that the market share of half-cells will grow from 5% in 2018 to almost 40% in 2028. Half-cell modules have solar cells that are cut in half, improving the efficiency and durability of the module. Traditional panels with 60 and 72 cells will have 120 and 144 cells, respectively. When solar cells are halved, their current is also halved, so resistive losses are reduced, and the cells can produce slightly more power. Smaller cells experience less mechanical stress, so cracking is less likely. Half-cell modules have higher output performance and are more reliable than traditional panels [151].

The beginning of the 21st century has seen a great abundance when it comes to new versions of seemingly long-known materials. Many of them have shown themselves to be extremely promising: graphene, carbon nanotubes, and, since 2012, perovskites [152].

Evolutionary developments in renewable energy are aimed at increasing the efficiency of solar panels, creating devices that are lighter, thinner, and closer to perfection in general. At the present stage of the development of microelectronics to solve the problem of heat removal and the production of highly efficient solar cells, the main hopes for a technological solution are pinned on graphene. Researchers at the Massachusetts Institute of Technology have found an alternative replacement for expensive materials in solar cells using organic materials with graphene electrodes. Batteries made using graphene show excellent efficiency results. The two-dimensional structure of graphene endows it with amazing properties of flexibility and transparency. Future developments aim to create thinner, more flexible, and more stable solar panels in the future.

Solar energy for a century and a half has made a qualitative leap from the household level (energy supply of buildings and outdoor lighting, equipment for surface buoys and signs, children’s toys, parking lots with electric charging stations, cars, and other vehicles) to space development. The pace of development of the alternative energy industry served as an impetus for the intensification of innovation activities, as well as the creation of new high-tech industrial sectors. The activity of each sector of the economy solves the problems of a technological, economic, and environmental nature in the field of alternative energy:

- Primary sector: mining and collection of minerals (in particular: copper, silicon, tellurium, cadmium, etc.)
- Secondary sector: manufacturing industry (aerospace, construction, etc.)
- Tertiary sector: the service sector in all sectors of the economy.
- Quaternary sector: intellectual services (technological progress).

Flexible solar modules have proven themselves in the field of medicine, communication systems, fire service, navigation and signal signs, police, army, maritime, search and rescue services, traffic surveillance and control systems on freeways, cinema, etc. Manufacturers of flexible panels, depending on the formulation of technical problems, make various modifications on the basis of solar modules of different types. The design and physical features expand the possibilities of their application: fixing onto objects with a complex configuration or non-smooth surface.

The relentless growth in demand for cheaper resources is driving the market towards the production of more efficient and cost-effective renewable energy sources. Factors limiting the absorption of sunlight by solar cells and reducing the efficiency of flexible solar cells can be dust, dirt, or bird droppings. A promising solution in the field of solar energy is the use of flexible solar panels, which is due to their geometric and physical parameters.

## 7. Key Findings and Future Implications

Thus, the following key findings of this review can be highlighted:

- Solar panel diversity: the review paper revealed a diverse landscape of solar panel technologies, including monocrystalline, polycrystalline, thin-film, and emerging third-generation solar cells. Each technology exhibited distinct advantages and limitations, impacting factors such as efficiency, cost, and manufacturing complexity.
- Material influence on efficiency: the study underscored how the choice of photovoltaic material profoundly influences solar panel efficiency. Monocrystalline silicon exhibited high conversion efficiencies due to its uniform crystal structure, while thin-film materials like cadmium telluride and copper indium gallium selenide demonstrated cost advantages but comparatively lower efficiencies.
- Bandgap engineering: photovoltaic materials with tunable bandgaps, such as perovskites and tandem solar cells, showcased the potential to optimize energy absorption across the solar spectrum. The manipulation of bandgaps allowed for enhanced performance in various lighting conditions.
- Material stability and durability: the review highlighted the importance of material stability in solar panel longevity. Emerging materials like perovskites demonstrated impressive efficiency gains but often faced challenges related to degradation under environmental stressors. Extensive research focused on enhancing stability to ensure long-term performance.
- Defect management and passivation: effective passivation techniques were identified as essential for minimizing defects at material surfaces and interfaces. Silicon-based solar cells employed techniques like hydrogenation, while emerging materials leveraged tunnel oxide passivated contacts (TOPCon) to reduce recombination losses and improve charge carrier extraction.
- Multijunction and tandem solar cells: multijunction and tandem solar cells were identified as effective strategies to improve overall efficiency by stacking different materials with complementary absorption spectra. These configurations demonstrated the potential to exceed the efficiency limits of single-junction solar cells.
- Cost reduction and scalability: the study emphasized the significance of advancing photovoltaic materials with abundant and non-toxic constituents, fostering cost-effective production and widespread scalability. The integration of advanced manufacturing techniques like roll-to-roll processing showcased potential for reducing material and production costs.
- Environmental impact: the environmental impact of photovoltaic materials was a central concern. The review paper discussed the importance of sustainable sourcing, recycling methods, and reducing toxic components to align with broader environmental goals.

When it comes to future implications, the following points can be considered:

- Advancements in material stability and durability: As the adoption of flexible solar panels continues to expand, the need for enhanced durability and long-term stability becomes paramount. Future research efforts should concentrate on developing materials that can withstand a variety of environmental stressors, such as UV radiation, temperature fluctuations, and moisture exposure. By engineering materials with improved stability, solar panels can maintain their performance over extended lifetimes, leading to greater economic viability and reduced maintenance requirements.
- Integration into urban infrastructure: The concept of solar-integrated urban landscapes holds immense promise for decentralized energy generation. Future research should focus on optimizing flexible solar panels for integration into existing and new architectural structures, facades, and even wearable devices. This integration not only presents opportunities to generate energy from underutilized surfaces but also contributes to sustainable urban planning, making clean energy generation a ubiquitous part of daily life.
- Innovations in manufacturing techniques: achieving cost-effective production methods without compromising efficiency is a persistent challenge in the solar industry. Researchers and engineers should explore novel manufacturing techniques, such as roll-to-roll printing, additive manufacturing, and continuous deposition processes, to enable large-scale production of flexible solar panels. By streamlining production processes, the cost barriers associated with these advanced materials can be reduced, making them more accessible for widespread deployment.
- Environmental impact and circular economy: as the world embraces renewable energy, considerations of the environmental impact of solar panel materials, production, and end-of-life management become increasingly relevant. Future research should delve into the development of sustainable material alternatives and explore recycling methods to ensure a circular economy approach. Addressing the environmental aspects of flexible solar panel technology will be instrumental in minimizing its ecological footprint and aligning with broader sustainability goals.
- Multi-functionality and energy storage integration: The integration of energy storage capabilities within flexible solar panels holds promise for a more seamless energy supply, enabling power generation even when sunlight is unavailable. Future research should explore ways to incorporate energy storage technologies, such as flexible batteries or supercapacitors, directly into the panel structure. This integration would enhance the versatility and reliability of solar panels, making them even more attractive for diverse applications.
- Global accessibility and energy equity: to truly harness the potential of flexible solar panels, research should not only focus on technological advancements but also on making these technologies accessible to underserved communities and remote regions. Future efforts should prioritize the development of low-cost, easy-to-install solar solutions that can provide clean energy to areas with limited infrastructure. Bridging the energy gap through innovative solar technologies has the potential to empower communities and contribute to global energy equity.
- In conclusion, the future of flexible solar panels and photovoltaic materials is teeming with possibilities and challenges that require multidisciplinary collaboration and innovative thinking. By addressing issues related to durability, scalability, cost, integration, sustainability, and accessibility, researchers and industry stakeholders can drive the transformation of solar energy from a niche technology to a pervasive and integral part of our sustainable energy landscape.

## 8. Conclusions

To conclude, this review paper provided a detailed portrayal of the present status of flexible solar panels and photovoltaic materials. The outcomes emphasize the prospective impact of burgeoning materials, notably graphene, perovskites, and organic compounds, in elevating the efficiency, malleability, and steadfastness of solar panels. These strides in innovation hold the potential to forge solar energy solutions that are not only lighter and more efficient but also remarkably adaptable across a spectrum of applications.

## Figures and Tables

**Table 1:** Main characteristics of solar cells based on CdTe [43,44,45,46].

Material Structure	S, cm^2^	Uoc, mB	Jsc, mA/cm^2^	FF, %	Eff-cy: %	Company
ss/ITO/CdS/CdTc//Cu/Au	0.191	790	20.10	69.4	11.0	IEC
ss/SnO_2_/CdS/CdTe	0.824	840	20.66	74.0	12.8	NREL
ss/SnO_2_/CdS/CdTe	0.313	783	24.98	62.7	12.3	Photon Energy
ss/SnO_2_/CdS/CdTe	0.3	788	26.18	61.4	12.7	Photon Energy
ss/SnO_2_/CdS/HgTeGa	1.022	736	21.9	65.7	10.6	SMU
MgF_2_/ss/SnO_2_/CdS//CdTe/C/Ag	1.047	843	25.09	74.5	15.8	Univ. South Florida
ss/SnO_2_/CdS/CdTe/Ni	1.068	767	20.93	69.6	11.2	AMETEX
ss/SnO_2_/CdS/CdTe	0.08	745	22.1	66.0	10.9	Georgia Tech.
MgF_2_/ss/SnO_2_/CdS/CdTe	1.115	828	20.9	74.6	12.9	Solar Cells Inc.
Ss/SnO_2_/CdS/CdTe/Cu/Au	0.114	815	17.61	72.8	10.4	Univ. Toledo
CdTe		807			12.7	BP Solar

**Table 2:** Important photovoltaic material parameters and their descriptions.

Full Name	Designation	Description
Efficiency	η	Efficiency is a crucial parameter and represents the ability of a solar cell to convert sunlight into electricity. It is the ratio of the electrical power output to the incident solar power. Higher efficiency means more effective energy conversion.
Open-Circuit Voltage	Voc	The voltage across the terminals of a solar cell when no external load is connected. It indicates the maximum voltage the solar cell can generate under illumination, and it is a key factor in determining the cell’s efficiency.
Short-Circuit Current	Isc	The current through the solar cell when its terminals are short-circuited. It represents the maximum current the cell can produce under full sunlight exposure.
Fill Factor	FF	The ratio of the maximum power output of the solar cell to the product of Voc and Isc. It gives insight into how effectively the solar cell utilizes its maximum power point.
Maximum Power Point	Pmax	This is the point on the current–voltage curve of the solar cell where the product of current and voltage is at its highest value. Pmax represents the maximum electrical power output the solar cell can generate.
Voltage at Maximum Power	Vmpp	The voltage at which the solar cell operates to produce the maximum power output.
Current at Maximum Power	Impp	The current at which the solar cell operates to produce the maximum power output.
Shunt Resistance	Rsh	Represents the resistance in parallel with the solar cell, which allows some current to bypass the cell. Higher Rsh values indicate lower leakage current and improved efficiency.
Series Resistance	Rs	Represents the resistance in series with the solar cell, reducing the voltage across the terminals. Lower Rs values lead to better performance.
Temperature Coefficient	-	Solar cell performance is influenced by temperature changes. The temperature coefficient describes how much the efficiency or other parameters change with temperature variations.
Spectral Response	-	Solar cells respond differently to different wavelengths of light. The spectral response curve shows how efficiently a solar cell converts light of various wavelengths into electricity.
Bandgap	Eg	The energy difference between the valence and conduction bands of the material. It determines the range of wavelengths that a solar cell can absorb and convert into electricity.
External Quantum Efficiency	EQE	Quantifies the efficiency with which a solar cell converts photons of different wavelengths into electrons. It provides insight into the solar cell’s performance across the solar spectrum.
Degradation and Stability	-	Over time, solar cells can experience performance degradation due to environmental factors, such as exposure to sunlight, temperature, and humidity. Understanding the stability and degradation mechanisms is crucial for long-term performance.

**Table 3:** The main characteristics of some solar cells based on silicon.

Material Structure	S, cm^2^	Uoc, mB	Jsc, mA/cm^2^	FF, %	Eff-cy	Date	Company
c-Si	4.00	709	40.9	82.7	24.0	9/94	UNSW
c-Si	45.7	694	39.4	78.1	21.6	4/94	UNSW
c-Si	22.1	702	41.6	80.3	23.4	5/96	UNSW
mc-Si	1.00	636	36.5	80.4	18.6	12/91	Georgia Tech.
mc-Si	100	610	36.4	77.7	17.2	3/93	Sharp
tf-Si	240	582	27.4	76.5	12.2	3/95	Astro Power
tf-Si	4.04	699	379	81.1	21.1	8/95	UNSW
a-Si:H	1.06	864	16.66	71.7	103	10/90	Chronar
a-Si:H	0.99	886	17.46	70.4	10.9	9/89	Glass tech.
a-Si:H	1.0	887	19.4	74.1	12.7	4/92	Sanyo
a-Si:H	1.08	879	18.8	70.1	1 1.5	4/87	Solarex
ITO/c-Si/a-Si	1.0	644	39.4	79.0	20.0	9/94	Sanyo
a-Si:H	1.0	891	19.13	70.0	12.0	9/94	Solarex
a-Si:H	1.0	923	18.4	72.5	12.3	9/94	Fuji-Elect.
a-Si/a-Si/a-SiGe		2320	7.3	73.0	12.4	9/94	Sumitomo
a-Si:H	1.0	887	19.4	74.1	12.7	9/94	Sanyo
a-C/a-SiML/a-SiC/a-Si	1.0	936	19.6	71.8	13.2	9/94	MutsuiToatsu
a-C/a-Si/a-SiC/a-Si	1.0	909	19.8	73.3	13.2	9/94	MutsuiToatsu
ITO/a-Si:H/a-SiGe:H	0.28	1621	1 1.72	65.8	12.5	1/92	USSC/Cannon
a-Si/k-Si	0.03	1480	16.2	63.0	15.0	9/94	Osaka Univ.
a-SiC/a-Si	1.0	1750	8.16	71.2	10.2	9/94	Solarex
a-Si/a-Si	1.0	1 800	9.03	74. I	12.0	9/94	Fuji
a-SiC/a-SiGe/a-SiGe	1.0	2290	7.9	68.5	12.4	9/94	Sharp
a-Si/a-Si/a-siGe	1.0	2550	7.66	70.1	13.7	9/94	ECD/Sovonics
p-a-SiO:H/a-Si:H/n-a-Si:H	1.0	899	18.8	74.0	12.5	9/94	Fuji-Elect.
ITO/a-Si:H/Si:H/a-siGe	0.27	2541	6.96	70	12.4	2/88	ECD
a-Si:H/a-Si:H/a-SiGc:H	1.00	2289	7.9	68.5	12.4	12/92	Sharp
a-Si/CuInSe_2_		871 432	16.4 17.4	72.0 68.0	10.3 5.3	9/94	ARCO
a-Si/mc-Si		917 575	10.4 30.2	76.0 79.2	7.25 13.75	9/94	Osaka Univ.

**Table 4:** Difference in temperature dependence of various PVMs [138].

PVM Types	MSW-180	Pramac Luce MCPH P7LM	BYD	Stion SN 130
Technology	Mono-Si, bilateral	α-Si/µ-Si	Multi-Si	CIGS
Voltage Temperature Coefficient, B/°C	−0.152	−0.004	−0.0034	-
Current Temperature Coefficient, AKC	0.00044	0.0006	0.00045	-
Temperature Coefficient of Power, Bt/°C	−0.005	−0.0025	−0.0047	−0.0045

## Data Availability

Data sharing not applicable.
